# Advancing Care for Older Adults Through Health Information Technology: An Interdisciplinary Approach

**DOI:** 10.1093/geroni/igaf122.377

**Published:** 2025-12-31

**Authors:** Zhang Zhang, Nancy Schoenborn, Thomas Cudjoe

## Abstract

Technology holds transformative potential to enhance healthcare delivery for older adults by improving access, efficiency, and patient-clinician communication. However, challenges persist such as heterogeneity across population groups in technology adoption, stakeholder priorities, and ethical concerns about transparency. This symposium addressed these gaps through four interdisciplinary studies, which provide insights into telehealth, patient portals, and artificial intelligence (AI) in the care of older adults. The first study examined telehealth’s role in expanding preventive care access and promoting early detection of dementia via Medicare Annual Wellness Visits (AWVs). Telehealth adoption was associated with an increase in AWVs. The second study found that among older adults, patients who send the most secure messages via the patient portal are more likely to be white, have a higher comorbidity burden, and exhibit greater patient portal and healthcare use, which require equitable digital access and tailor patient portals to better support diverse needs. The third study adopted a mixed-methods approach to test ChatGPT’s ability to identify nonpatient authors of dementia-related portal messages. Interviews with people with dementia and caregivers revealed conditional acceptance of AI-drafted messages, contingent on clinician review and explicit disclosure of AI involvement. The fourth study delved into stakeholder decision-making regarding AI adoption in healthcare. Interviews with older adults, caregivers, clinicians, payers, and investors revealed shared key factors in decision-making (e.g., usability, value) but divergent emphasis on cost. Our discussant will explore implications for care innovation and approaches to address the barrier to technology, including strategies to ensure inclusive implementation across diverse aging populations.

